# Significance of microRNA targeted estrogen receptor in male fertility

**Published:** 2014-02

**Authors:** Alireza Abhari, Nosratollah Zarghami, Vahideh Shahnazi, Abolfazl Barzegar, Laya Farzadi, Hadi Karami, Sepideh Zununi Vahed, Mohammad Nouri

**Affiliations:** 1 Department of Biochemistry and Clinical Laboratories, Faculty of Medicine, Tabriz University of Medical Sciences, Tabriz, Iran; 2 Women’s Reproductive Health Research Center, Alzahra Hospital, Tabriz, Iran; 3 Research Institute for Fundamental Sciences (RIFS), University of Tabriz, Tabriz, Iran; 4 Department of Biotechnology, Faculty of Advanced Medical Science, Tabriz University of Medical Sciences, Tabriz, Iran

**Keywords:** Fertility, Has-mir-100, Has-let-7b, MicroRNA

## Abstract

***Objective(s): ***Estrogen receptor-alpha (ERα) mediates estrogen action in regulation of different levels of the hypothalamic-pituitary-testis axis. It has a key role in spermatogenesis. Estrogen receptor alpha knock-out (ER koα) male mice were infertile and severe impairment in spermatogenesis and seminiferous tubules was observed. Recently, it has been reported that microRNA (miRNA) mir-100 and let-7b were predicted to target ERα gene. MiRNA are small, endogenous, single stranded RNA molecules that regulate gene expression and have been implicated in various disease states. It has been proved that some miRNAs expression is tissue- and disease-specific, giving potential for identifying miRNAs as a diagnostic tool.

***Materials and Methods:*** In this study, the change in the expression levels of mir-100, let-7b and ERα expression levels were evaluated in oligospermic infertile patients (n=43) compared to control fertile subjects (n=43). After washing and separating sperms, total RNA was isolated and then cDNA was synthesized. The expression levels of mir-100 and let-7b and ERα were evaluated by real time PCR.

***Results: ***Mir-100, let-7b levels were significantly higher than those in control group (*P*=0.008 and *P*=0.009, respectively). We have found that, ERα level was significantly decreased in comparison with normal group (*P*< 0.0001).

***Conclusion: ***Changes in mir-100, let-7b and ERα expression levels in oligospermic patients may be associated with the susceptibility and progression of infertility. The results of this study indicate that miRNA can have a key role in spermatogenesis and might have a diagnostic and prognostic value in men infertility.

## Introduction

Infertility is defined as a failure to bring baby after 12 or more months of consistently intercourse without contraception (1). More than 15% of couples are infertile, about 40% of which is related to male factors. Oligospermia and asthenospermia are common causes of infertility in males, however the molecular mechanisms causing these effect is not entirely clear. The causes are known in less than 35% of these cases and more than 60% related to genetic disease with unknown molecular mechanisms. In Oligospermia, the number of spermatozoa is reduced and in asthenospermia abnormality in sperm motility is seen (2). About 60-75% of infertility cases are idiopathic with or without abnormal semen analysis (3). It was seen that in bulls, spermatozoa with normal motility and morphology, the fertility of some bulls was reduced which may be due to molecular defects in the sperm cells (4).

More than hundreds of genes are involved in spermatogenesis. Molecular and cellular integrity of sperm cells is important for fertilization any deletion or mutation in the sequence of genes and inappropriate gene expression cause disorder in spermatogenesis and fertility (5, 6). Estrogen has a positive impact on the function of sperm by stimulating capacitation and fertilizing ability (7). As Also, it has a key role in modulating male reproductive tract. Cellular signaling of estrogen is mediated through the estrogen receptors (ERα) (8) that are present throughout the male reproductive tract and spermatozoa. ERα mediates estrogen action in regulation of different levels of the hypothalamic-pituitary-testis axis. ERα has an essential role in male fertility, it was proved that ER knockout (Era-/-) mice become infertile (9).

ERα gene expression is regulated by small noncoding RNAs (microRNA). Previous studies showed that mir-100 and let-7b were predicted to target ERα gene (10, 11). MicroRNAs (miRNAs) were first detected in human spermatozoa by Ostermeier *et al *they are abundant in spermatozoa (12) but their function in spermatogenesis and fertilization is unknown. miRNAs are small (18-25 nucleotides) noncoding regulatory RNAs which negatively regulate gene expression (13). They participate in the designation of cell fate, embryonic development, control of growth, differentiation, and the death of cells (14). There is a number of miRNA expressed in male mouse germ cells. miRNAs are involved in regulation of gene expression during mitotic, meiotic, and post-meiotic stages of spermatogenesis (15). Impaired biogenesis of miRNAs disrupts spermatogenesis and causes infertility in male mice (16). In the present study, we investigated the expression levels of mir-100, let-7b, their common target gene (ERα) and their correlation with oligospermic and normospermic control in men.

## Materials and Methods

***Study design***

From infertile men (n=723) referred to Alzahra Infertility Center, Tabriz, Iran (age 27.5 + 4.8 years), 43 oligospermic infertile patients were selected. The written consent of the subjects was done according to medical ethics. Control samples (n=43) wereselected from normal volunteers who had a baby in the last two years and their semen analysis was normal. Two months before sampling, none of the control subjects nor patients treated with the drug and they didn’t have intercourse 3-5 days before sampling. This research was approved by the Ethics Committees of Tabriz medical University, Tabriz, Iran.

***Exclusion criteria***

The volunteers with infertile partner, infection in the genital tract, autoimmune disorders, reproductive tract abnormality, smoking, and alcohol and drug consumption were excluded from the study.

***Isolation of spermatozoa from seminal fluid***

Semen samples were collected in a sterile container and incubated at 37°C for 30 min to get the fluid. Then, semen analysis was performed according to WHO guidelines (2010). Sperms were purified by Goodrich methods (17). In brief, the samples were washed two times in 1×PBS buffer solution, then somatic cells were absent in SCLB solution ( 0.1% SDS, 0.5% TX-100 in DEPC water). The cells were counted, if somatic cells were present the process was repeated. Finally, the solution was frozen at –80°C.

***RNA isolation***

Total RNA was isolated using Exiqon miRCURY RNA isolation kit (Exiqon, Denmark) according to the manufacturer instructions. Quantity and quality of the isolated RNA was measured by Nanodrop 1000 (NanoDropND-1000spectrophotometer; Thermo Fisher Scientific, Waltham, MA). Total RNAs were reversed to cDNA using LNA universal RT miRNA PCR kit (Exiqon, Denmark). Briefly, 20 ng of total RNA was reverse transcribed. cDNA synthesis was performed by thermal cycler (Eppendorf, Germany) with the following parameter values; 60 min at 42°C, 5 min at 95°C and immediately cooled to 4°C until use.

***Real-time PCR analysis***

Quantitative real-time reverse transcriptase-PCR was carried out using the Corbett Rotor-Gene 6000 Real-Time PCR system (Qiagen, Germany). miRNAs quantification was performed using MiRCURY LNA Universal RT microRNA PCR system (Exiqon, Denmark). Mir-16 was used as the endogenous control miRNA. The relative expression level of ERα was measured by qPCR with primers (ERα: 5′-CCACATCAGTCACATGAGTAA-3′ and 5′-GTTCCATCAGCATCTACAG-3′) using SYBR Green PCR Kit (Qiagen, Germany). The expression levels were normalized to β-actin as housekeeping gene with the following primers (5′-TGGACTTCGAGCAAGAGATG-3′ and 5′-GAAGGAAGGCTGGAAGAGTG-3′). The reactions were performed in triplicate.

***Statistical analysis***

Statistical analysis was performed using SPSS software (version 18). The results were expressed as mean ± SD. Relative expression level of genes were calculated by the 2^DDCq^ model (18). Unpaired Student's t-test was used to analyze the differences in gene expression between oligospermic and control group. Correlation analysis was performed using the Spearman rank correlation test. In all analysis, *P*-value < 0.05 was considered as significant.

## Results

***Expression level of mir-100, let-7b and ERα in oligospermic and control group***

**Figure 1:**
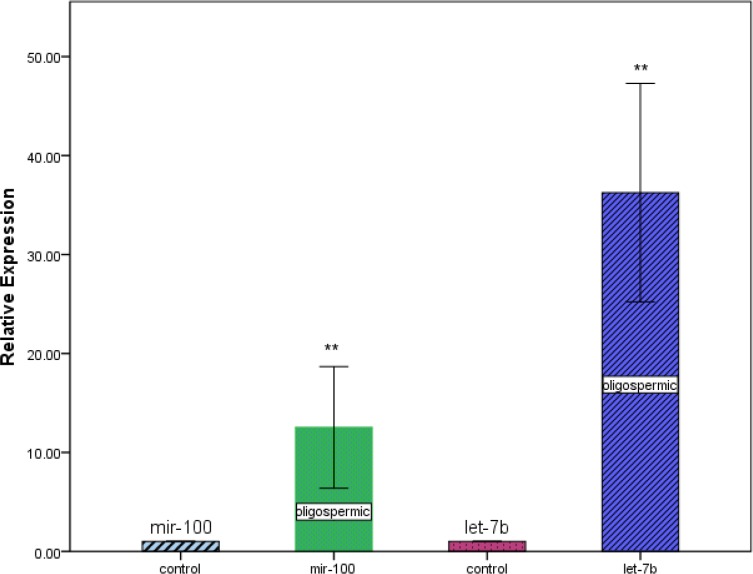
Relative expression levels of mir-100 and let-7b in oligospermic and control group. **P<0.01 compared with control group

**Figure 2:**
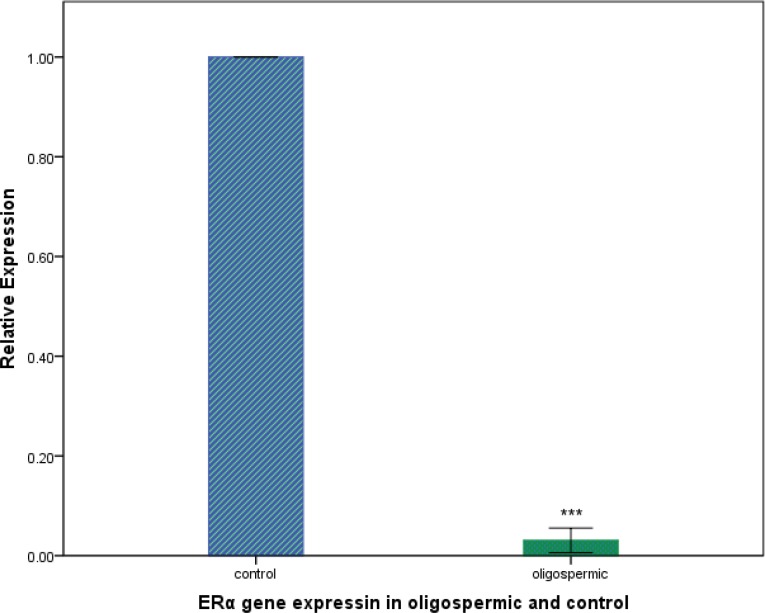
Relative expression levels of the estrogen receptor alpha (ERα) gene in control and oligospermic group. ***P<0.001 compared with control group

We determined the expression levels of mir-100, let-7b and ERα in oligospermic and control group. By real-time quantitative RT-PCR analysis, we found that, expression levels of mir-100 and let-7b were much higher in oligospermic than control group (*P*=0.008 and *P*=0.009, respectively, Figure 1). Inversely, expression level of ERα was significantly lower in oligospermic than control group (*P*<0.0001, Figure 2).

***Correlation between expression levels of ERα and seminal plasma parameters***

Correlation between expression levels of ERα and semen were analyzed using Spearman^׳^s rank correlation test (Table 1). Expression levels of ERα were strongly and positively correlated with those of sperm count, quick progressive, slow progressive and normal morphology (Spearman^׳^s correlation coefficient; +0.863, +0.723, +0.875 and +0.642, respectively) and negatively correlated with immotile (Spearman^׳^s correlation coefficient; -0.691).

***Correlation between expression levels of miRNAs and seminal plasma parameters***

Relationship between expression levels of miRNAs and semen parameters, such as volume, sperm count, quick progressive, slow progressive, non-progressive, immotile, normal morphology and pH was evaluated using Spearman^׳^s rank correlation test (Table 1). Expression levels of miRNAs were not significantly correlated with those of other semen parameters.

## Discussion

Spermatogenesis is an intricate process of germ cell development in which many genes are involved. Any defect in genes expression or their regulation, disrupts spermatogenesis and causes infertility (19, 20). miRNAs regulate gene expression by modification of special mRNA translation. Few studies have been conducted on miRNAs function in spermatogenesis and male fertility (21). In a study conducted on non-obstructive azoospermic infertile patients, significant change in miRNAs expression was seen compared to fertile control men (22). In that study, Lian *et al *showed that miRNAs have a regulatory role in spermatogenesis. In our study, we investigated mir-100, let-7b and their common target ERα gene expression in oligospermic infertile patients and compared them with normospermic fertile control by real-time PCR methods. Our result showed that mir-100 in oligospermic was significantly over expressed compared to control group. We also demonstrated that high mir-100 expression was associated with significant decreases in ERα gene expression level in oligospermic group. It was proved that mir-100 targets ERα gene and directly sets the level of the ERα gene (10, 23). Zhao *et al *reported significant inverse association between expression level of let-7b and ERα in breast cancer. Similarly, in the present study, we have shown that expression level of let-7b was increased unlike ERα expression, which was decreased. Recent studies demonstrated that, let-7b has an inhibitory effect on cell proliferation (11, 24). Guarducci *et al* showed that ERα promoter polymorphism were inversely associated with sperm count (25). As our results indicated, inhibition of germ cell proliferation by reducing the expression of ERα gene by let-7b is possible. In the male reproductive tract, there are higher levels of ERα in the efferent ductules (region of the male tract) than female reproductive system, it occupy one third of epididymis. It shows the importance of ERα in the male reproductive system and fertility. ERα regulates fluid reabsorption in the epididymis and is responsible for maintaining fluid osmolality and pH (26). In our study, reduced ERα expression was associated with little change in semen pH. Gunawan *et al *showed that a polymorphism in the coding region of ERα in exon 1 was related to sperm motility (27). Recent findings are consistent with our results. We proved that expression level of ERα in oligospermic was significantly down regulated compared to control group. Also, motility and pH in seminal fluid of oligospermic was lower than those in control. Our data showed that, spermatozoa with normal morphology was decreased in oligospermic group compared to control group. Moreover, our results showed significant positive correlation between the expression of ERα and the morphology. This is consistent with recent findings of Josepha and collegues. They proved that in ERαKO mice sperm maturation and capacity to fertilize were destroyed and contributed to infertility (28). ERα plays an important role in the balancing of sperm metabolism (29) and its dysfunction causes reduction of sperm density, sperm motility, and percentage of sperm with normal cell morphology (30). ERα in the acrosome of the spermatozoa is more than in other sections, acrosome contains Lysis enzymes which puncture the outer coat of the egg and allow the infiltration of sperm (31). Acrosome dysfunction impairs oocyte fertilization and causes male infertility (32). Oligospermic patients have a high frequency of defective sperm zona pellucid interaction (33, 34) and according to our finding, decrease in ERα may cause acrosome dysfunction.

**Table 1:** Correlation analysis among estrogen receptor alpha, mir-100, let-7b and seminal plasma parameters

Variable	Mir-100	Let-7b	ERα
Volume	0.469^a^	0.105	-0.092
	0.171^b^	0.772	0.799
Sperm count	-0.328	0.297	0.863*
	0.354	0.403	0.001
Quick progressive	-0.157	0.375	0.723*
	0.663	0.285	0.018
Slow progressive	-0.406	0.093	0.875*
	0.243	0.696	0.0009
Non-progressive	-0.105	0.377	0.551
	0.772	0.281	0.098
Immotile	0.171	-0.367	-0.691*
	0.630	0.296	0.026
Normal morphology	0	0.453	0.642*
	1	0.188	0.045
pH	0.151	0.333	0.345
	0.676	0.346	0.328

^a ^Spearman correlation coefficient

^b ^Spearman s rank correlation test

* *P*<0.05 is considered significant

**Table 2:** Comparison of sperm parameters of control and oligospermic group

Variable	Control (n=43)	Oligospermic(n=43)	*P*-value
Volume ml	3.616 ± 0.1744	3.035 ± 0.2344	0.0498
Sperm count × 10^6^sperm/ml	75.74 ± 2.840	9.465 ± 0.5834	<0.0001
Quick progressive %	15.00 ± 1.581	2.000 ± 2.000	0.0009
Slow progressive %	25.00 ± 2.739	12.00 ± 1.225	0.0025
Non progressive %	31.00 ± 3.674	16.00 ± 2.449	0.0094
Immotile %	27.00 ± 2.000	72.00 ± 3.391	<0.0001
Normal morphology %	26.53 ± 0.9188	10.47 ± 0.5213	<0.0001
pH	7.353 ± 0.03840	7.316 ± 0.07306	0.6607
Abstinence day	3.800 ± 0.2494	3.600 ± 0.3055	0.6182

*P<0.05 is considered significant

Control, sperm count≥15 × 10^6^sperm/ml; Oligospermic, sperm count < 15× 10^6^ sperm/ml

It is possible that miRNA interfere with spermatogenesis through other genes. Possible targets for let-7b are SRC1, PTEN, MEST, AKT1 and AKT2. Both PTEN and AKT1 are common targets for mir-100 and let-7b. SRC1 (Steroid receptor co-activator 1) is a transcriptional co-activator of many transcription factors involving nuclear receptor. It is a transcriptional partner of a co-activator of ERα. SRC is involved in signaling pathways that lead to self-renewal or differentiation of spermatogonial stem cells (35). AKT1 is a serin/theronin kinase enzyme that has been proved to be the mediator of cellular growth, proliferation, survival, and metabolism in various cell types (36). Kim *et al *showed that in Akt2-/- male mice, apoptotic sperms in null mice were more than wild-type mice, and sperm motility and concentration were significantly lower in the null sperm (37). PTEN (phosphatase and tensin homologue deleted on chromosome ten) plays special roles in different cellular processes, including cell transformation, survival, proliferation, migration and mediate the differentiation of germ cells (38). Defect in MEST function or processing was correlated with low sperm counts. MEST hyper methylation was seen in idiopathic infertile men with sperm morphology below 5% normal spermatozoa and progressive sperm motility below 40%. It is a biomarker of sperm quality (39, 40).

## Conclusion

We have defined efficacy of mir-100, let-7b and ERα in oligospermic infertile patients. Our study obtained more information on the molecular mechanism of infertility, and their possible regulatory role in spermatogenesis and fertilization. miRNAs might have a diagnostic and prognostic value in oligospermic infertile men.
